# Diagnosing acute myocarditis in the emergency department—advancing cardiac MRI with a focus on low-field MR applications

**DOI:** 10.3389/fradi.2025.1652004

**Published:** 2026-01-08

**Authors:** Ehsan Karimialavijeh, Latika Giri, Eduardo Baettig, Muhammad Umair

**Affiliations:** 1 Kaiser Permanent, Central Valley, CA, United States; 2 Kathmandu University School of Medical Sciences, Dhulikhel, Nepal; 3 Department of Radiology, Hospital Clínico Universitario de Valencia, Valencia, Spain; 4 Columbia University Irving Medical Center, New York, NY, United States

**Keywords:** abbreviated protocol, acute myocarditis, artificial intelligence (AI), cardiac magnetic resonance (CMR), emergency department, low-field MRI, parametric mapping

## Abstract

Acute myocarditis is an inflammatory condition of the myocardium, often triggered by viral infections, autoimmune diseases, or toxins. It can lead to arrhythmias, heart failure, and sudden cardiac death. Early and accurate diagnosis is crucial for timely management and preventing complications. It poses a significant diagnostic challenge in emergency departments (EDs) due to nonspecific symptoms, overlapping features with conditions like acute coronary syndrome, and limitations of conventional diagnostics. Cardiac magnetic resonance imaging (CMR) is the gold standard for noninvasive diagnosis, using the 2018 Modified Lake Louise Criteria (mLLC). However, high-field CMR (1.5–3T) faces barriers in EDs, such as longer scan times, higher cost, lack of accessibility, and contraindications in patients with implantable devices, severe kidney disease, or hemodynamic instability. Low-field MRI (<1.5T) offers advantages in portability, safety, and cost while reducing susceptibility artifacts. Recent advances in AI-driven image reconstruction (e.g., LoHiResGAN, U-net) address low signal-to-noise ratios, enabling cine imaging, strain analysis, and parametric mapping at 0.55T. Studies show that low-field CMR can detect subclinical myocarditis and predict outcomes, with ECV measurements at 0.55T strongly correlating with 1.5T (*r* = 0.91), demonstrating comparable reliability. By integrating low-field CMR into ED protocols, clinicians can improve early detection of occult myocarditis, guide risk stratification, and reduce long-term morbidity and healthcare costs. Standardization of imaging workflows and AI-enhanced protocols will further bridge diagnostic gaps, particularly in resource-limited settings. This review highlights low-field CMR's potential to redefine acute myocarditis management, balancing diagnostic precision with practicality in emergency care.

## Introduction

Acute myocarditis, an inflammatory disorder of the myocardium often triggered by viral infections (e.g., coxsackievirus, adenovirus), autoimmune reactions, or toxins (e.g., chemotherapy, alcohol), poses a significant diagnostic challenge in emergency departments (EDs) (1, 2). Its clinical manifestations range from mild, self-limiting symptoms (e.g., fatigue, chest pain) to life-threatening complications such as fulminant myocarditis, arrhythmias, and sudden cardiac death (3, 4). The nonspecific nature of symptoms—overlapping with conditions like acute coronary syndrome (ACS) or Takotsubo cardiomyopathy—often leads to misdiagnosis and delayed treatment (5, 6).

Traditional tools, including electrocardiography (ECG), cardiac biomarkers (e.g., troponin), and echocardiography, lack sensitivity and specificity for its diagnosis (7–9). While ECG may show ST-segment elevation in 70% of cases, troponin sensitivity varies widely (64%–100%), and echocardiography frequently fails to detect early myocardial dysfunction in up to 75% of patients (8). These limitations are particularly pronounced in subclinical or atypical cases, where symptoms like flu-like malaise or mild chest discomfort are dismissed as benign (10). Undetected myocarditis can progress to dilated cardiomyopathy (DCM), chronic heart failure, or sudden cardiac death (11). Long-term management of complications, such as heart failure, imposes substantial economic burdens. Heart failure treatments have a wide range of cost-effectiveness, measured by cost per quality-adjusted life year (QALY). Medications such as beta-blockers, Angiotensin-Converting Enzyme inhibitors, and angiotensin receptor blockers have high QALY and are cost-effective, but advanced therapies (e.g., ventricular assist devices, transplantation) are expensive (12). Despite limited direct cost data, the global incidence of myocarditis has risen significantly, increasing by 62% from 1990 to 2019, with approximately 1.27 million cases reported annually (13). Over the same period, myocarditis-related deaths surged by 65%, reaching 324,490, highlighting their growing public health burden (14).

The 2018 mLLC established CMR as the gold standard for noninvasive diagnosis of myocarditis. This criterion integrates native T1 mapping, extracellular volume (ECV) calculation, T2 mapping, short tau inversion recovery (STIR), and late gadolinium enhancement (LGE) to detect myocardial edema, necrosis, and fibrosis. In a key validation cohort, it demonstrated a sensitivity of 87.5% and a specificity of 96.2%, though these values can vary with patient population and timing of imaging. Major guidelines, including the European Society of Cardiology, give CMR a class I recommendation for the evaluation of suspected myocarditis (15). Additionally, parametric mapping techniques, such as ECV quantification, allow risk stratification by identifying diffuse fibrosis, which has been linked to adverse outcomes (16).

Recent international guidelines have further refined the diagnostic and management pathways for myocarditis. The 2025 European Society of Cardiology (ESC) Guidelines emphasize early risk stratification, structured use of cardiac magnetic resonance imaging, and selective endomyocardial biopsy in high-risk presentations. Similarly, the 2024 American College of Cardiology (ACC) Expert Consensus Decision Pathway provides a pragmatic framework for emergency and inpatient evaluation, highlighting CMR as the preferred noninvasive modality when myocarditis is suspected after exclusion of obstructive coronary disease (17, 18).

Despite its diagnostic superiority, high-field CMR (1.5–3T) faces significant barriers in EDs, including prolonged scan times (60–90 min), contraindications in hemodynamically unstable patients or those with ferromagnetic implants, and limited accessibility due to cost and infrastructure (19, 20). Low-field systems offer advantages in the assessment of a broader range of cardiac diseases, particularly in patients with cardiac devices, where they produce fewer susceptibility artifacts compared to high-field MRI due to their lower magnetic field strength (21–23).

## CMR in acute myocarditis diagnosis

### Current literature on CMR for acute myocarditis

We conducted a comprehensive search to identify studies on the use of CMR in myocarditis across multiple databases, including PubMed, Embase, Medline, Web of Science (WOS), and Cochrane, covering publications from 2017 to 2025. The search covered publications from January 2017 to March 2025 to capture the most recent advancements, particularly in low-field MRI. The search strategy incorporated various keyword combinations related to MRI modalities (“MRI,” “magnetic resonance imaging”), cardiac conditions (“cardiac,” “heart,” “cardiovascular,” “myocarditis”), and applications (“utility,” “feasibility,” “diagnostic value,” “technical challenges,” “clinical use”). A specific focus was given to low-field, portable, and point-of-care MRI, particularly in emergency settings (“emergency,” “urgent,” “ED,” “ER”). Boolean operators (AND, OR) and field-specific filters (e.g., Title/Abstract) were used to refine results.

Inclusion criteria encompassed original research articles, clinical trials, and case reports involving human subjects, published in English, that investigated CMR for myocarditis diagnosis, risk stratification, or outcome prediction. Reviews, editorials, and non-English publications were excluded. The initial search yielded a broad set of articles, which were screened by title and abstract for relevance. The full text of potentially eligible articles was then reviewed to confirm their alignment with the review's focus, particularly on emergency and point-of-care applications. Data on study design, patient population, CMR protocols, diagnostic performance, and outcomes were extracted from the included studies.

**Figure 1:**
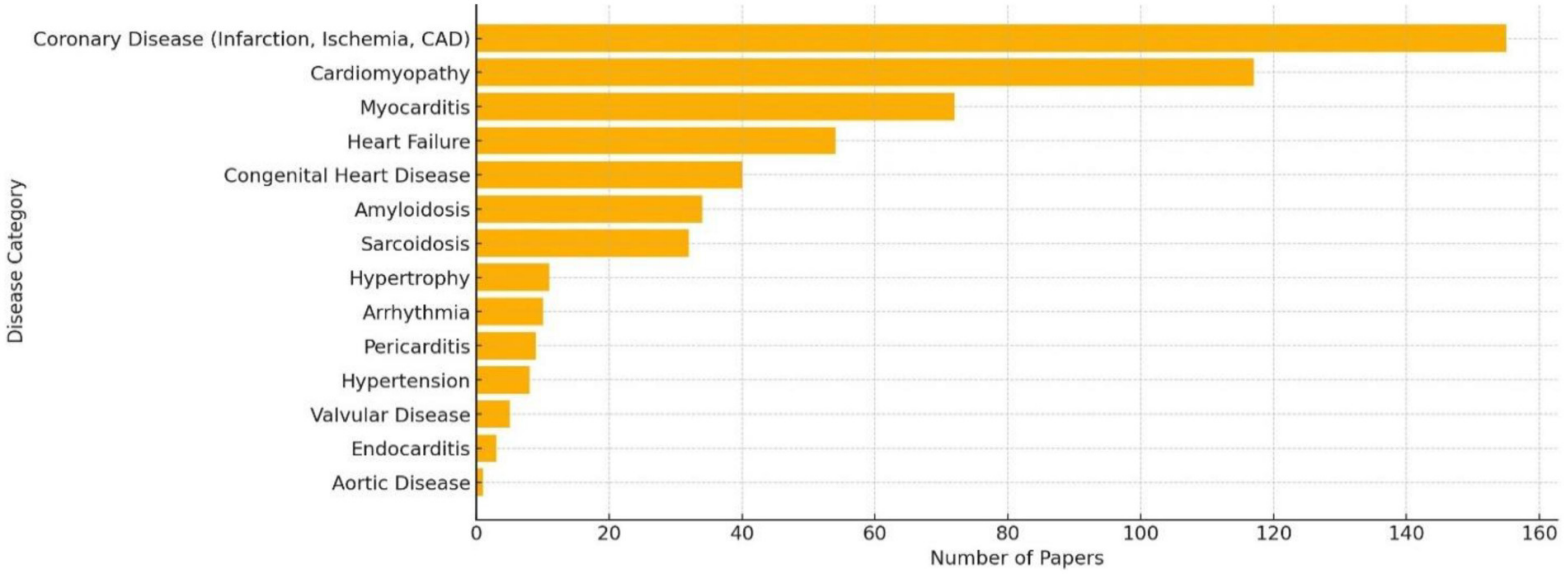
Number of CMR papers by disease category (2017–2025).

Our review of the publications about CMR in cardiac diseases from 2017 to 2025 reveals that myocarditis is the third most common pathology studied in research among any CMR diagnoses, after coronary disease and cardiomyopathy (Figure 1).

**Figure 2:**
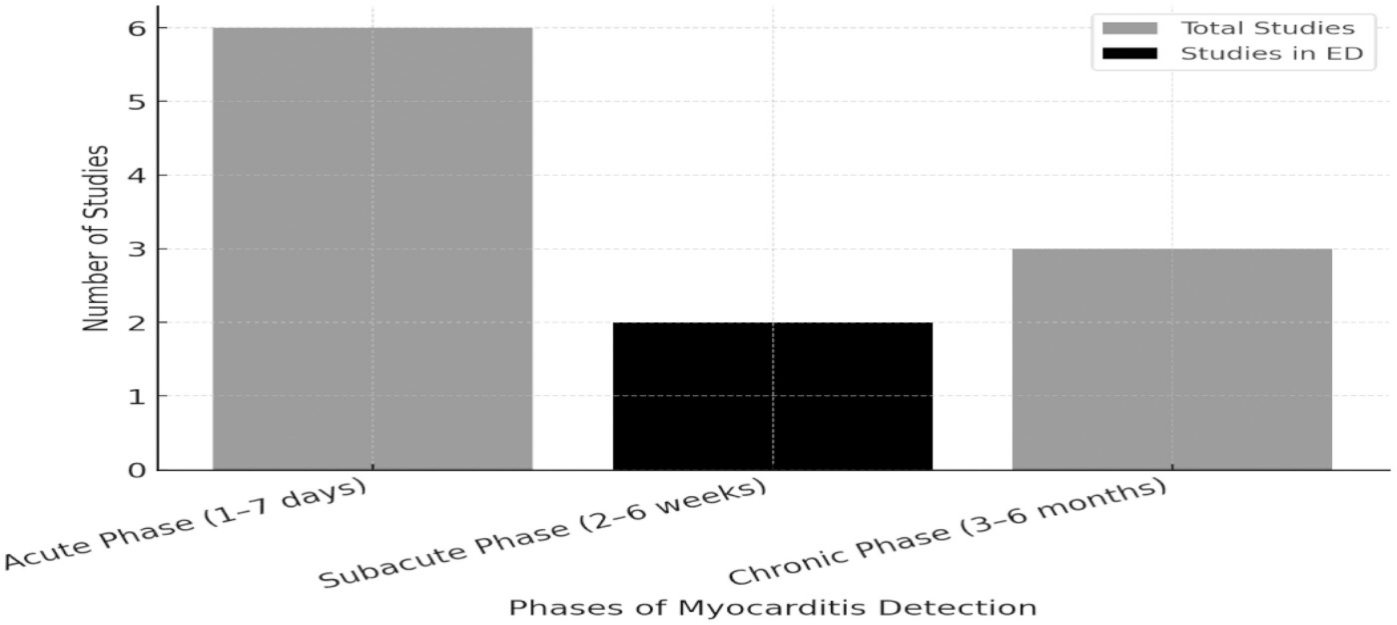
Applications of CMR in myocarditis based on a literature review of studies published between 2017 and 2025. This figure presents six studies investigating the use of CMR in the acute phase of myocarditis. Among these two studies, Alotaini et al. (25) and Sasa et al. (72) specifically assess CMR in the ED setting, evaluating its feasibility and diagnostic performance for real-time clinical decision-making in acute myocarditis cases.

This trend highlights the growing importance of CMR in cardiac imaging research, particularly in the use of CMR for myocarditis management. Among these studies, CMR is widely used for the diagnosis, monitoring, and prognosis of myocarditis (Figure 2).

Although CMR has been utilized in different stages of myocarditis, only two studies were conducted in the ED setting (24, 25). However, the second study conducted by Alotaibi et al. (25), focused on recurrent myocarditis presented to the ED. This indicates that, despite CMR's excellent diagnostic yield and its paramount importance in the accurate diagnosis of myocarditis, its utility in the ED is not well established.

### Missed myocarditis: challenges in diagnosing atypical and subclinical myocarditis

The clinical and imaging features of myocarditis often overlap with other cardiac conditions, such as myocardial infarction (MI), Takotsubo cardiomyopathy, and sarcoidosis (26), making accurate differentiation challenging, especially in patients with elevated troponin levels and nonspecific ECG changes (27). While coronary angiography can rule out obstructive coronary disease, it does not assess myocardial inflammation, underscoring the need for CMR in these cases (15).

Patients with Takotsubo cardiomyopathy frequently present with acute chest pain, dyspnea, ECG changes (including ST-segment elevation or T-wave inversion), and elevated cardiac troponin—features that are clinically indistinguishable from myocarditis or ACS at presentation. Importantly, Takotsubo cardiomyopathy is often triggered by emotional or physical stress and predominantly affects postmenopausal women, but these epidemiologic clues are not sufficiently specific to reliably guide early diagnosis in the ED (28).

Conventional diagnostic tools further contribute to this overlap. Echocardiography may demonstrate regional wall motion abnormalities in both Takotsubo cardiomyopathy and myocarditis, while coronary angiography may be normal in both conditions, particularly in myocarditis and Takotsubo syndrome without obstructive coronary disease. As a result, relying solely on symptoms, biomarkers, ECG findings, and initial imaging may lead to misclassification and delayed or inappropriate management. Cardiac magnetic resonance imaging plays a critical role in resolving this diagnostic ambiguity. CMR can reliably differentiate Takotsubo cardiomyopathy from myocarditis (15, 26).

Atypical acute myocarditis, presenting with chest pain but normal ECG and troponin levels, is frequently misdiagnosed as acute coronary syndrome (ACS), leading to delays in treatment (29). Conventional imaging modalities such as echocardiography, cardiac CT, and point-of-care echocardiography (POCUS) lack the sensitivity and specificity required for a definitive diagnosis (30, 31). Characteristic subepicardial and mid-wall late gadolinium enhancement (LGE) patterns are highly specific for myocarditis and correlate with disease severity and prognosis (32). Furthermore, CMR has been instrumental in identifying myocardial inflammation in post-Coronavirus Disease-2019 (COVID-19) and vaccine-associated myocarditis, highlighting its expanding role in clinical practice (33).

With refined imaging protocols and diagnostic criteria, CMR is increasingly recognized as the gold standard for noninvasive myocarditis diagnosis, offering superior accuracy compared to conventional imaging and even endomyocardial biopsy (34). As demonstrated for other causes of chest pain, exploring the integration of CMR into emergency settings could, pending further validation, significantly enhance early diagnosis and management, potentially reducing misdiagnosis and invasive procedures (35).

### Role of mLLC in diagnosing myocarditis

**Figure 3:**
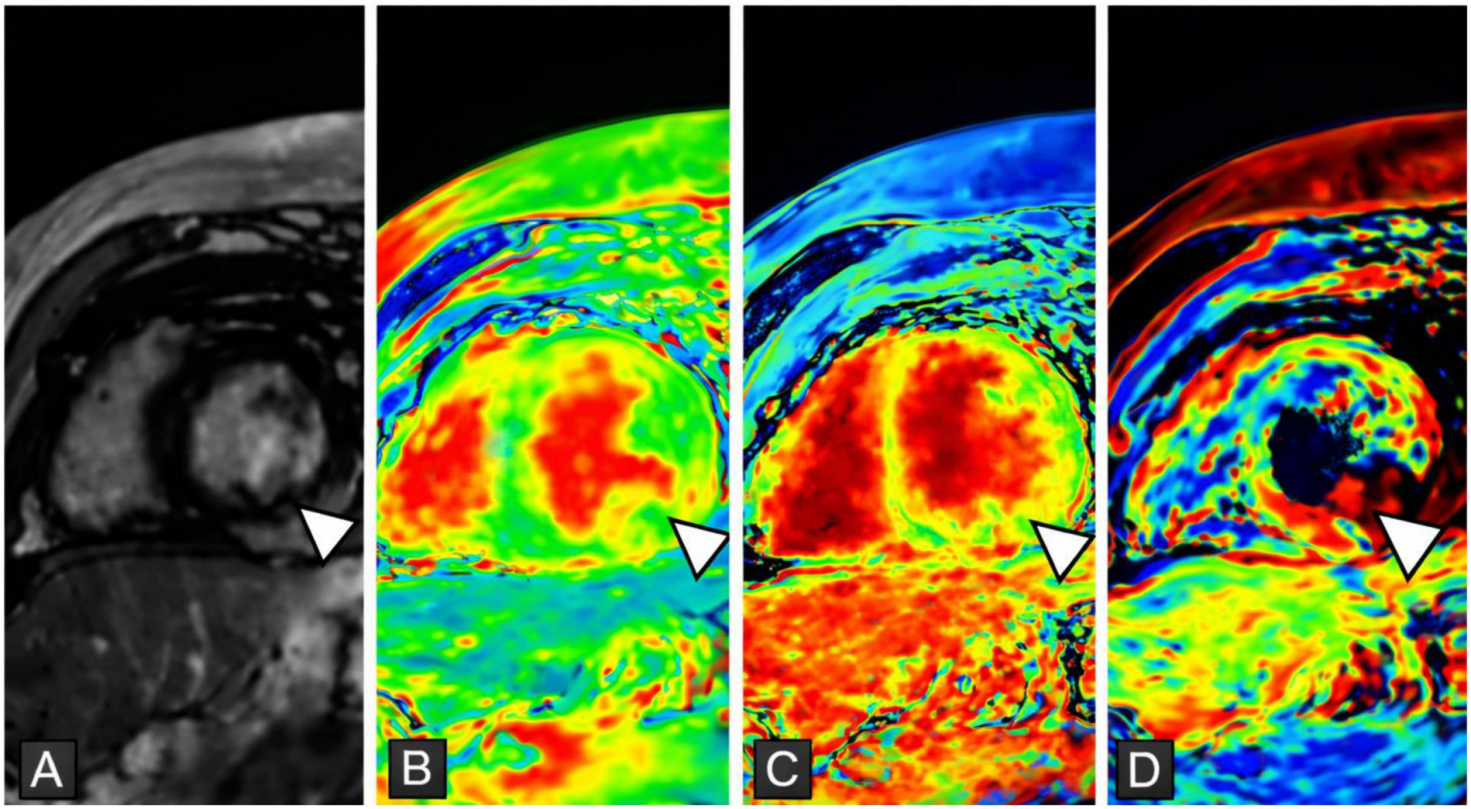
Eighteen-year-old male hospitalized for myocarditis. CMR images showing subepicardial involvement in the inferolateral wall, demonstrated by LGE, T1 native mapping, ECV and T2 mapping (arrowheads). **(A)** LGE image demonstrating subepicardial non-ischemic enhancement in the inferolateral left ventricular wall (arrowhead), typical of acute myocarditis. **(B)** Native T1 mapping showing focally elevated T1 values in the same inferolateral region, reflecting myocardial injury and inflammation (arrowhead). **(C)** ECV map demonstrating increased ECV in the inferolateral wall, consistent with expansion of the extracellular space due to edema and inflammatory infiltration (arrowhead). **(D)** T2 mapping revealing regional T2 elevation in the inferolateral myocardium, indicating active myocardial edema (arrowhead). *© Dr Eduardo Baettig, Hospital Clínico Universitario de Valencia, Spain*.

The Lake Louise Criteria, first introduced in 2009, revolutionized CMR myocarditis diagnosis by incorporating T2-weighted imaging (for edema), early T1-based LGE sequences (for hyperemia), and late LGE sequences (for necrosis and fibrosis) (36). The 2018 mLLC established a systematic approach to diagnosing myocarditis using CMR by detecting myocardial inflammation through both T1- and T2-based techniques (37) (Figure 3). The T2-based criterion identifies myocardial edema, a marker of acute inflammation, which can be assessed using traditional T2-weighted STIR sequences or the more sensitive quantitative T2 mapping (38). The T1-based criterion confirms additional myocardial abnormalities through LGE in a nonischemic pattern, such as subepicardial or mid-myocardial enhancement, or through quantitative techniques like native T1 mapping and ECV quantification, which allow for precise tissue characterization (38). According to the mLLC, the presence of at least one T2 abnormality and one T1 abnormality is required for a definitive diagnosis of myocarditis (37–39).

### Parametric mapping in myocarditis diagnosis

Quantitative mapping techniques have significantly improved the accuracy of myocarditis detection by enabling quantitative assessments of myocardial tissue properties, particularly in identifying early and borderline cases of myocarditis (40). Table 1 summarizes the key parametric mapping techniques, T1/T2 mapping and post-contrast T1 mapping-derived ECV quantification, with their unique properties in the detection and evaluation of myocarditis.

**Table 1:** Key quantitative mapping techniques in myocarditis diagnosis.

Imaging technique	Key features	Distinctive properties
T1 mapping (without contrast)	Elevated in acute myocarditis due to edema and necrosis	Useful for patients with renal impairment (when only native T1 is performed), it reflects myocardial edema and necrosis (43, 44)
Post-contrast T1 Mapping & ECV	Elevated ECV and shortened post-contrast T1 mapping in myocarditis due to necrosis and extracellular matrix expansion	Assesses myocardial damage and disease progression, both in the early stages due to gadolinium leakage and in the later stages as fibrosis (37)
T2 Mapping (without contrast)	Elevated in acute myocardial inflammation due to edema	Highly sensitive for acute injury, in the form of edema, distinguishes acute from chronic forms (45)

The inclusion of novel parametric mapping techniques, native T1 mapping, T2 mapping, and ECV in the 2018 mLLC has significantly enhanced diagnostic yield compared to the original criteria. In a validation study, the mLLC achieved a sensitivity of 87.5% (95% CI: 73.9%, 94.5%) and a specificity of 96.2% (95% CI: 81.1%, 99.3%), outperforming the original LLC criteria, which had a sensitivity of 72.5% (95% CI: 57.2%, 83.9%) (41). It is important to note that these performance metrics are derived from specific cohorts and may vary in different clinical settings. Several studies have further explored the combination of these parametric mapping techniques, resulting in improved sensitivity and specificity, with Li et al. reporting a high diagnostic performance when combining native T1 and T2 mapping, showing a diagnostic accuracy of 92% for myocarditis detection (42). When used together, parametric mapping techniques provide a comprehensive, noninvasive approach to diagnosing myocarditis, improving both diagnostic accuracy and the ability to monitor disease evolution over time (37).

## CMR for risk stratification and outcome prediction

Subclinical myocarditis is particularly challenging to diagnose in both emergency and outpatient settings due to the low sensitivity of conventional methods. Ng et al. reported that although only 44% of recovered COVID-19 patients had elevated troponin levels, cardiac MRI revealed subclinical myocardial inflammation in 56% of cases, manifested as elevated T1 and/or T2 mapping values, with or without nonischemic LGE (10). Similarly, Wang et al. demonstrated that CMR using a 1.5T MRI had a sensitivity of 87.5% and specificity of 96.2% in detecting myocardial abnormalities in patients with normal ECG and troponin, reinforcing its value in identifying occult myocarditis in their study population (30).

A study by Puntmann et al. showed how CMR can make therapeutic decisions among patients with nonspecific symptoms like fatigue, mild chest pain, or arrhythmias (46). Subepicardial and mid-wall enhancement patterns on CMR, which are characteristic of myocarditis, were observed in 86% of cases in a study by Schwab et al. (32). CMR extends beyond diagnostic capabilities, offering critical risk stratification and prognostication in myocarditis and related cardiac pathologies (26). LGE extent is a robust predictor of adverse cardiac events, including arrhythmias, heart failure, and mortality (33, 41).

Studies by Radunski et al. and Biesbroek et al. have demonstrated LGE's predictive power for progression to dilated cardiomyopathy (4, 47). Elevated T1 and T2 values, as shown by Isaak et al., also correlate with adverse long-term outcomes (2). ECV mapping further refines risk stratification by quantifying diffuse microscopic-level fibrosis (48), while feature-tracking strain analysis identifies early deformation abnormalities predictive of adverse remodeling (40). In athletes, CMR's ability to detect LGE and T1/T2 abnormalities post-COVID-19, as highlighted by McKinney et al., is crucial for return-to-play decisions, mitigating the risk of sudden cardiac death (33).

Furthermore, artificial intelligence (AI) emerges as a powerful tool to enhance CMR's clinical impact. Studies by Papetti et al. and Wang et al. demonstrate the potential of AI for automated image segmentation and interpretation, significantly improving efficiency and accuracy in CMR analysis in controlled environment (49, 50). For example, Papetti et al. successfully utilized a convolutional neural network (CNN) model to segment the left ventricle (LV) and myocardial infarction scar (MIS) on a series of dark blood late gadolinium enhancement (DB_LGE) obtained from 144 patients using 1.5T CMR, improving speed compared to semiautomated techniques (53). Also, Wang et al. reported high performance (area under the curve of 0.988 ± 0.3% for screening and 0.991 ± 0.0% for diagnostic models) of their computerized model for CMR interpretation among 9,719 patients (49). These AI tools are currently exploratory and require regulatory approval and external validation before routine clinical use.

Preliminary studies suggest low-field CMR may have prognostic capabilities in myocarditis. Spieker et al. and Puntmann et al. demonstrated that elevated T2 and T1 values are predictors of adverse outcomes in myocarditis in post-COVID patients in their respective cohorts (51, 52).

Isaak et al. showed that transmural LGE at 0.55T correlates with a 3.5-fold increased risk of heart failure hospitalization over 5 years (53). Lin et al. further validated that low-field CMR achieves 92% agreement with high-field systems in LGE detection in a specific patient group, supporting the need for further investigation into its use in outcome prediction, particularly in resource-limited settings (54). Standardized longitudinal studies are now needed to establish low-field-specific prognostic thresholds and refine risk assessment strategies.

## Limitations of high-field CMR (1.5–3T) in emergency settings

High-field CMR systems face several limitations in ED settings, impacting their feasibility for acute patient management. Prolonged scan times (exceeding 60–90 min) can delay critical decision-making and are often unsuitable for hemodynamically unstable patients, particularly those with hypotension or arrhythmias. Patient cooperation is frequently compromised due to breath-holding requirements and motion artifacts, which are particularly problematic in acutely ill individuals (55). Additionally, implantable cardiac devices such as pacemakers and implantable cardioverter defibrillators (ICDs) limit CMR use due to risks of device heating or displacement (56).

Limited accessibility and high operational costs further restrict the widespread adoption of high-field CMR in rural or resource-constrained EDs. These challenges are compounded by maintenance expenses, personnel requirements, and the cost of gadolinium-based contrast agents (GBCAs) (57).

Claustrophobia and discomfort in narrow-bore scanners frequently lead to incomplete studies, especially in obese, anxious, or acutely symptomatic patients (55, 58). Additionally, susceptibility artifacts from metallic objects can degrade image quality (56).

Finally, infrastructure demands, including shielded rooms and stable power supplies, make high-field CMR impractical in overcrowded or outdated EDs (59). Lack of portability and protocol variability across institutions further complicates standardized integration into acute care workflows (60, 61).

## The future of myocarditis diagnosis through advances in low-field CMR

### Low-field MRI benefits

Unlike high-field MRI systems, low-field MRI provides a more accessible alternative in various clinical settings. Recent studies have demonstrated the feasibility of using portable low-field MRI for point-of-care neuroimaging, offering actionable results within minutes. This capability may significantly improve patient outcomes in time-sensitive scenarios such as traumatic brain injury, musculoskeletal imaging, and pulmonary pathologies (61–63).

### Low-field MRI in portable set up in ED

Studies such as those by Littlewood et al. and Lin et al. demonstrated the capability of low-field MRIs to perform advanced imaging techniques with acceptable diagnostic accuracy in motion-corrected, 3D LGE imaging and free-breathing scans (19, 64). Furthermore, low-field MRI systems can be used in rural or remote EDs, as seen in Lin et al.'s report of a portable 0.5T MRI scanner for diagnosing myocarditis in underserved regions (54).

Recent pilot studies demonstrate the initial feasibility of low-field CMR in EDs, addressing gaps in clinical validation. For example, Littlewood et al. successfully implemented a 0.55T MRI system in an ED workflow, performing motion-corrected 3D LGE imaging in hemodynamically stable patients with suspected myocarditis (19). Their protocol achieved diagnostic accuracy comparable to that of 1.5T systems, with 88% concordance in detecting subepicardial fibrosis and edema, while reducing scan times to 25–35 min (19). Similarly, Lin et al. validated free-breathing, AI-reconstructed low-field CMR (0.35T) in rural EDs, reporting 92% agreement with high-field CMR for diagnosing acute myocarditis in patients with nondiagnostic echocardiography (64). This study highlighted low-field CMR's utility in triaging high-risk patients (e.g., troponin-negative chest pain) while minimizing claustrophobia-related scan failures (<5% vs. 15% in 1.5T systems) (64). As shown in Table 2, key parametric measurements such as ECV quantification and T1 mapping in selected studies exhibit correlations between low-field (0.35–0.55T) and high-field (1.5–3T) systems, but these findings are vendor-, sequence-, and site-specific (65). For instance, Lin et al. used free-breathing T2 mapping, whereas Littlewood et al. prioritized abbreviated LGE protocols (19, 64). Standardization efforts, such as the MYOFLAME-19 trial (43), aim to establish consensus workflows for low-field CMR in acute care.

**Table 2:** Diagnostic accuracy of low-field vs. high-field CMR in myocarditis.

Parameter	Low-field CMR (0.35–0.55T)	High-field CMR (1.5–3T)	Study (year)
ECV correlation	*r* = 0.91 vs. 1.5T	Reference standard	Kaushal et al. (2024) (65)
T1 mapping	Feasible (AI-enhanced)	AUC 0.92 for myocarditis	Ferreira et al. (2018) (15)
Sensitivity (mLLC)	85% (AI protocols)	87.5% (mLLC criteria)	Wang et al. (2024) (49)
Specificity (mLLC)	91% (site-calibrated)	96.2% (mLLC criteria)	Kaushal et al. (2024) (65)
LGE detection	88% concordance with 1.5T (motion-corrected)	Gold standard for necrosis/fibrosis	Littlewood (2024) (19)

Kaushal et al. (65) demonstrated that ECV measurements at 0.55T strongly correlate with 1.5T (*r* = 0.91), validating low-field reliability for fibrosis quantification. High-field benchmarks: Sensitivity/specificity from Luetkens et al. (38) reflect the 2018 mLLC criteria. Low-field sensitivity: estimated from Wang et al. (49) on AI-enhanced CMR interpretation.

Both the 2025 ESC Guidelines and the 2024 ACC Expert Consensus emphasize cardiac magnetic resonance imaging as the cornerstone of non-invasive myocarditis diagnosis, particularly when the clinical presentation is atypical or when biomarkers are inconclusive. The ACC pathway specifically highlights the emergency department as a key decision point, recommending early CMR in patients with chest pain, elevated troponin, and non-obstructive coronary arteries (17, 18).

### Patient safety and comfort

One of the major advantages of low-field MRI is its reduced magnetic field strength, which mitigates safety concerns, especially for patients with ferromagnetic implants. With lower magnetic forces, risks like tissue heating, device displacement, or malfunction are minimized (56). Additionally, low-field MRI systems produce fewer susceptibility artifacts compared to high-field MRI, enhancing diagnostic accuracy. A study also highlighted the quieter operation of low-field MRI systems, which improves patient comfort, especially for those sensitive to noise or has anxiety during imaging procedures. 84% of patients rated the noise levels of 0.55T MRI as “better” or “much better” than a 1.5T MRI (55). These patient safety parameters are of utmost importance in ED patients who are acutely symptomatic and anxious while receiving urgent care.

### Avoidance of claustrophobia

Many low-field MRI systems come with open configurations, which can be more comfortable for patients with claustrophobia or obesity. However, some studies reported a higher incidence of claustrophobia in open MRIs as well, which could be attributable to prior negative MRI experiences (58).

### Enhancing image quality with AI

AI plays a significant role in overcoming the challenge of low signal-to-noise ratio (SNR) in low-field MRI systems, thus improving image quality. AI-driven models such as LoHiResGAN (Low- to High-Resolution Generative Adversarial Network) have shown significant improvements in image quality, especially in brain MRI (66). Moreover, AI models like CNN and U-net have been used to enhance scan timing, image reconstruction, resolution, myocardial segmentation, quantification, and T1/T2 mapping. These tools are not yet standardized or widely approved for clinical use.

### Advanced imaging techniques with low-field MRI

Several studies have confirmed the feasibility of advanced imaging techniques in low-field MRIs in research contexts. Kaushal et al. reported equivalent and reliable diagnostic correlations between ECV measurements at 0.55 and 1.5T, while Varghese et al. demonstrated the feasibility of advanced imaging techniques such as phase-contrast imaging and T1/T2 mapping at 0.35T (67). Additionally, Doerner et al. highlighted the potential of strain analysis at low-field strengths for detecting minor myocardial changes, adding diagnostic value in cases like myocarditis, where clinical symptoms can be nonspecific (68).

### Cost-effectiveness of low-field CMR

Low-field MRI has the potential to reduce economic costs significantly, making it an attractive option for emergency and resource-limited settings. Estimated purchase prices are often cited in the range of $150,000–$500,000, which is 70%–80% lower than high-field systems (often >$1 million, up to $3 million for premium 3T systems), offering substantial upfront savings. Installation costs may also be reduced by eliminating the need for heavily shielded rooms, potentially saving $200,000–$500,000 in infrastructure upgrades (69). Operationally, low-field CMR may lower annual maintenance costs ($20,000 vs. $100,000–$150,000 for high-field) by eliminating the need for heavily shielded rooms, potentially saving $200,000–$500,000 in infrastructure upgrades (19). Its portability could enable deployment in standard ED bays without facility renovations (22). A detailed micro-level cost-effectiveness analysis (CEA) based on device utilization, scan time, personnel, and patient outcomes would be further needed to confirm the potential savings.

## Challenges and future directions in low-field CMR implementation

### Limitations of low-field MRI and technological improvements to address limitations

Despite the potential advantages, low-field CMR still faces challenges such as low spatial resolution and low SNR, which may hinder the ability to distinguish between different tissue types. For instance, myocardial fibrosis and subtle edema may be difficult to diagnose due to these limitations. Recent innovations, such as the optimization of pulse sequences for low-field systems and advanced motion correction algorithms, are actively being developed to overcome these limitations (59). Additionally, techniques like compressed sensing, parallel imaging, and machine learning-based approaches are being shown in research settings to improve SNR and spatial resolution, making low-field CMR more suitable for clinical use (66, 69).

### Standardization of imaging protocols

Standardized imaging protocols are crucial for enhancing the diagnostic accuracy and reproducibility of low-field CMR in myocarditis. A recent study using AI-driven approaches to fine-tune T2 mapping sequences highlights this need (70). AI-driven tools, such as motion correction and noise reduction algorithms, have the potential to improve image quality and ensure consistency across clinical applications, but they require standardization and validation. To guide future research and development for low-field CMR in emergency and outpatient settings, we propose a structured imaging workflow that integrates examples of AI-enhanced techniques. The following table (Table 3) outlines a proposed standardized low-field CMR protocol for research applications in the evaluation of suspected myocarditis:

**Table 3:** Proposed applicable low-field CMR protocol for suspected myocarditis.

Sequence	Parameters	Acquisition planes	Scan duration	AI Tool	Purpose
T2 Mapping	Free-breathing, motion-corrected	Short-axis stack	4–5 min	U-net (52)	Quantify edema
Native T1 Mapping	Free-breathing, motion-corrected	Short-axis stack	4–5 min	U-net (52)	Quantify baseline tissue characterization
Cine Imaging	Real-time, free-breathing	Short-axis stack + 2/3/4-chamber views	5–7 min	Compressed sensing (71)	Assess wall motion/function
3D LGE Imaging	3D IR, motion correction	Short-axis stack + 2/3/4-chamber views	10–12 min	LoHiResGAN (64)	Detect focal fibrosis/scar
Post-contrast T1 Mapping (for ECV calculation)	Free-breathing, motion-corrected	Short-axis stack	4–5 min	Automated segmentation (49)	ECV calculation (post-processing)

All times refer to sequence acquisition time only; post-processing (e.g., ECV calculation) is not included in these durations. Free-breathing sequences are used to accommodate patients unable to hold breath; acceleration is achieved via compressed sensing and/or parallel imaging.

This protocol leverages AI for noise reduction (LoHiResGAN), motion correction (U-net), and rapid analysis, reducing total scan time to 35–45 min while maintaining diagnostic accuracy. For example, Littlewood et al. achieved 88% concordance with high-field CMR using a similar 28-minute protocol (19). Future efforts, such as the MYOFLAME-19 trial (66), aim to validate these workflows against endomyocardial biopsy and clinical outcomes, bridging the standardization gap in low-field CMR.

### Advancements in biomarkers and diagnostic sensitivity

Advanced biomarkers, such as strain analysis, T1/T2 mapping, and ECV quantification, are critical to expanding the clinical usefulness of low-field CMR. Studies have shown that low-field MRI systems can produce reliable results for assessing myocardial fibrosis and edema, with strong correlations observed between ECV measurements at 0.55 and 1.5T (71). AI-driven improvements in strain analysis, ECV quantification, and image reconstruction can further enhance the diagnostic sensitivity of low-field CMR.

### Regulatory and implementation considerations

The integration of AI tools into clinical workflows requires careful consideration of regulatory compliance (e.g., FDA, CE marking), reproducibility across different hospitals and patient populations, and robustness against artifacts. AI-based reconstruction and analysis tools are currently in the research stage and should be clearly distinguished as exploratory supports, not the primary basis for clinical decision-making. Prospective comparison with standard methods and external validation are essential before clinical adoption.

### A proposed research agenda for clinical implementation

To maximize the clinical impact of low-field CMR in emergency care and translate its potential into clinical reality, a structured research agenda and implementation pathway are essential. We propose a multi-faceted approach beginning with ED Prospective Multicenter Trials to compare the diagnostic accuracy, time-efficiency, safety, and cost-effectiveness of abbreviated low-field CMR protocols against standard care (e.g., echocardiography ± CT) for suspected acute myocarditis. Pilot studies suggest such an approach could reduce unnecessary admissions by 30%–40% by providing rapid, definitive diagnosis through motion-corrected T1/T2 mapping and LGE imaging (19, 54).

**Figure 4:**
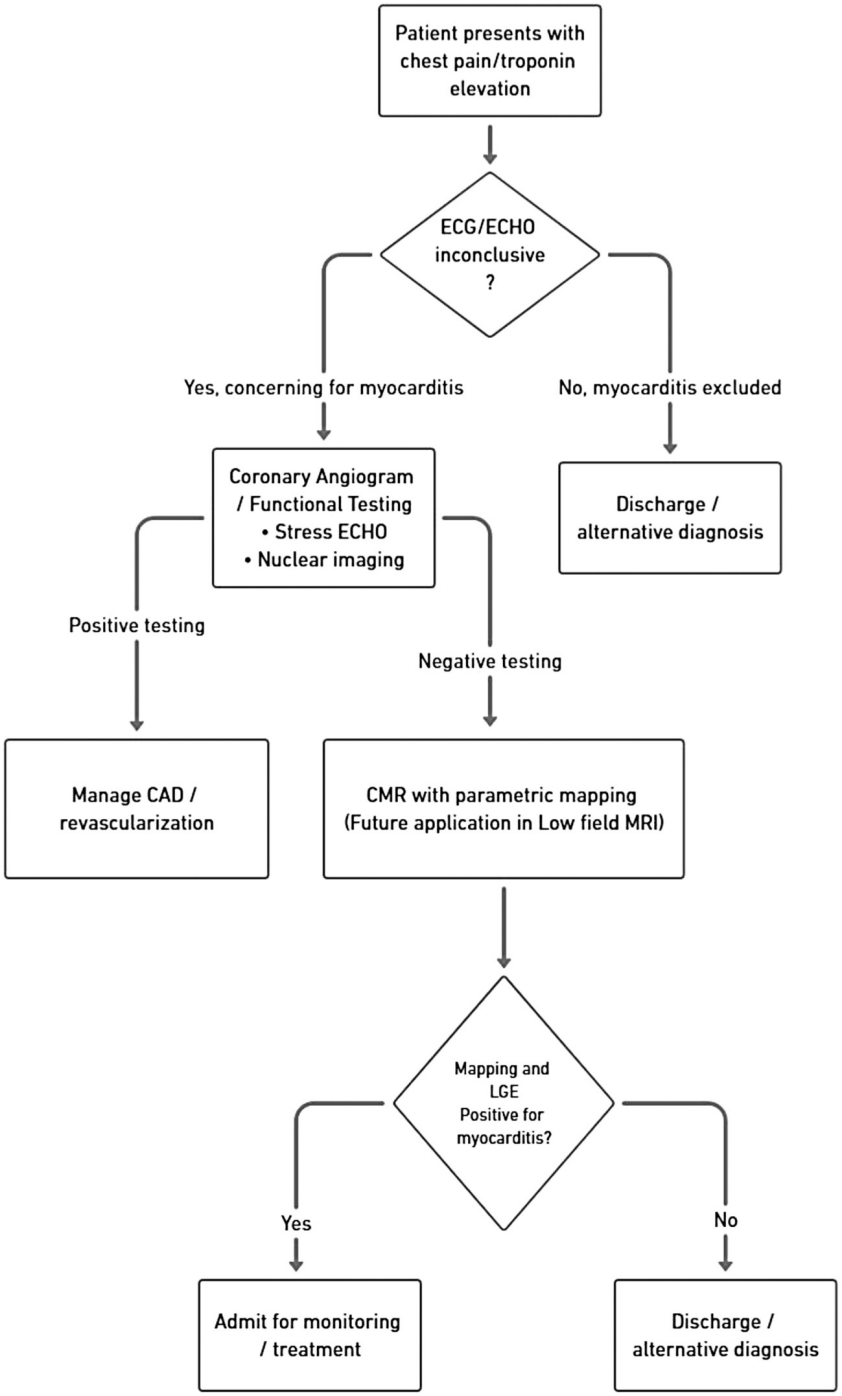
Proposed ED triage workflow integrating low-field CMR for suspected myocarditis. Rapid protocols enable same-day diagnosis, reducing unnecessary admissions.

Concurrently, systematic Image Quality and Artifact Evaluation in diverse patient subgroups (e.g., those with arrhythmia, obesity, or implants) are needed, alongside efforts to validate the diagnostic non-inferiority of Non-Contrast Protocols (such as Virtual Native Enhancement) compared to the standard LGE-based mLLC. To ensure reliability, the development of low-field-specific Quality Control and Assurance protocols, including phantom testing and drift monitoring for quantitative mapping is a critical priority. Finally, this research must culminate in the Standardization and Workflow Integration of consensus protocols for acute care. To guide this future implementation, we propose a structured triage pathway (Figure 4) that integrates rapid, protocol-driven low-field CMR into the ED evaluation of suspected myocarditis following inconclusive initial testing. This algorithm prioritizes patient safety through a mandatory pre-scan checklist assessing hemodynamic stability, renal function (e.g., eGFR >30 mL/min/1.73 m^2^ for contrast), pregnancy status, device compatibility, and procedure tolerance. Within this workflow, AI-based tools for reconstruction and analysis are positioned as exploratory supports for research purposes only, with final clinical decisions resting on standard, validated image interpretation until regulatory approval and broader clinical validation are achieved.

## Conclusion

Low-field CMR represents a promising technological advancement with the potential to enhance the diagnosis of acute myocarditis in emergency settings. Its inherent advantages in portability, safety, patient compatibility, and potential cost-effectiveness could help overcome the significant limitations of high-field CMR in the ED. Emerging data suggest that low-field CMR, particularly when augmented by investigational AI-driven reconstruction and parametric mapping, may detect tissue abnormalities consistent with myocarditis in selected settings. However, robust prospective evidence for its prognostic stratification and impact on patient outcomes remains limited.

Standardized protocols could reduce variability and facilitate broader adoption. Prospective multicenter feasibility and outcome studies are required before broad ED integration can be recommended. Key priorities for future research include validating diagnostic accuracy against clinical endpoints, establishing low-field-specific quantitative thresholds, developing robust non-contrast alternatives, and demonstrating cost-effectiveness in real-world emergency care. By addressing these challenges through a coordinated research agenda, low-field CMR could eventually fulfill its promise as a transformative tool for the early and accurate diagnosis of acute myocarditis, particularly in resource-constrained and point-of-care environments.
